# Oxidative stress as a driver of organelle cascade damage in neurological diseases

**DOI:** 10.3389/fnagi.2026.1892923

**Published:** 2026-07-22

**Authors:** Shiwang Li, Taijian Cao, Qiang Zhang

**Affiliations:** 1 School of Clinical Medicine, Qinghai University, Xining, Qinghai, China; 2 Qinghai University Affiliated Hospital, Xining, Qinghai, China; 3 Department of Neurosurgery, Qinghai Provincial People's Hospital, Xining, Qinghai, China

**Keywords:** inflammatory response, mitochondria, neurological diseases, organelles, oxidative stress

## Abstract

As a core driver in the pathological progression of neurological diseases, oxidative stress contributes to the onset and development of multiple disorders, including traumatic brain injury (TBI), Alzheimer’s disease (AD), Parkinson’s disease (PD), Huntington’s disease (HD), and amyotrophic lateral sclerosis (ALS), by inducing interconnected and bidirectional damage among mitochondria, endoplasmic reticulum, lysosomes, and the nucleus. This review systematically summarizes the oxidative stress-mediated inter-organelle crosstalk network: Mitochondria act as one of the earliest and central hubs, and their dysfunction (e.g., reactive oxygen species burst, calcium overload, and respiratory chain impairment) induces endoplasmic reticulum stress via ROS diffusion and calcium signaling disturbance. The disruption of endoplasmic reticulum calcium homeostasis further exacerbates mitochondrial damage, forming a vicious cycle. Lysosomes exhibit reduced membrane stability and impaired autophagic flux under oxidative stress, failing to clear damaged organelles and aggravating oxidative stress accumulation. Ultimately, oxidative stress signals are transmitted to the nucleus, resulting in DNA damage, aberrant epigenetic modifications, and activation of pro-inflammatory/pro-apoptotic genes, thereby accelerating disease progression. Notably, this organelle injury transmission is not a rigid unidirectional linear cascade; primary lysosomal or MAM defects can independently initiate the full organelle damage loop without preceding mitochondrial dysfunction. This review integrates current studies, clarifies context-dependent and disease-specific characteristics of organelle interactions, and discusses potential therapeutic strategies with critical consideration of translational challenges and limitations, providing a theoretical foundation for mechanistic research and clinical intervention of neurological diseases.

## 1 Introduction

Neurological diseases include traumatic, degenerative, and other types, with complex and interrelated pathological mechanisms. Organelle dysfunction induced by oxidative stress is a key common feature throughout the process. Oxidative stress refers to a pathological process in which the balance between the production and clearance of reactive oxygen species (ROS) is disrupted under stimulation by various pathological factors, leading to lipid peroxidation, protein oxidation, and DNA damage. In the nervous system, highly metabolic cells such as neurons are extremely sensitive to oxidative damage because they rely on efficient energy supply and precise organelle cooperation to maintain their functions.

This narrative review was completed via systematic literature searches conducted in the PubMed and Web of Science databases, covering publications published between 2020 and 2026. The literature retrieval was performed with reference to systematic review search criteria rather than full adherence to formal systematic review protocols. Eligibility criteria included peer-reviewed original articles and reviews focusing on oxidative stress, inter-organelle communication, and neurological diseases. Evidence quality was prioritized based on *in vivo*, time-course, and clinically relevant studies.

Mitochondria, known as the “powerhouses” of cells, generate adenosine triphosphate (ATP) through oxidative phosphorylation (OXPHOS) and are also the main source of ROS. The functional integrity of mitochondria is crucial for neuron survival. When stimulated by trauma, ischemia, protein aggregation, etc., mitochondria first exhibit functional abnormalities, manifested as decreased activity of respiratory chain complexes, collapse of membrane potential, massive release of ROS, and calcium overload. This process is considered the initiating event of oxidative stress-driven cascade damage (Tahmasebinia et al., 2025; Dong et al., 2024). For example, at 3 h after traumatic brain injury (TBI), selective peroxidation of mitochondrial-specific phospholipid cardiolipin (CL) occurs, which is earlier than the appearance of apoptotic markers (Bayir et al., 2007); in Alzheimer’s disease (AD), *β*-amyloid (Aβ) oligomers directly bind to mitochondrial membrane proteins, inhibiting respiratory chain function and inducing ROS production (Rai et al., 2020).

ROS released from mitochondrial damage attack the endoplasmic reticulum through diffusion, triggering endoplasmic reticulum stress. As the core organelle for protein folding and calcium storage, the endoplasmic reticulum depends on calcium signal interaction with mitochondria to maintain its homeostasis; This process is regarded as one of the critical initiating events of oxidative stress-mediated organelle damage (Tahmasebinia et al., 2025; Dong et al., 2024). Oxidative stress damages the structure and function of mitochondria-associated endoplasmic reticulum membranes (MAMs), leading to abnormal release of endoplasmic reticulum calcium stores, activation of the unfolded protein response (UPR), and further deterioration of mitochondrial damage via calcium overload, forming a bidirectional positive feedback loop of “mitochondria–endoplasmic reticulum” cross-injury (Thapak and Gomez-Pinilla, 2024; Ryan et al., 2020).

Lysosomes clear damaged organelles and abnormal proteins through the autophagy-lysosome system, serving as an important barrier to block cascade damage. However, oxidative stress can disrupt lysosomal membrane stability, reduce the activity of acid environment-dependent hydrolases, and cause obstruction of autophagic flux (Ma et al., 2023; Kiraly et al., 2025). For example, after TBI, increased lysosomal membrane permeability leads to the release of cathepsins into the cytoplasm, exacerbating mitochondrial damage (Courtes et al., 2020); in AD, cholesterol accumulation inhibits the recruitment of autophagy receptors, hindering mitochondrial clearance, and ultimately resulting in the accumulation of damaged organelles and toxic substances, continuously amplifying oxidative stress (Roca-Agujetas et al., 2021).

The nucleus, as the center of cellular genetic regulation, is one of the major terminal targets of oxidative stress-mediated cascade damage. Oxidative stress directly damages DNA (such as accumulation of 8-hydroxydeoxyguanosine), interferes with epigenetic modifications (such as histone acetylation and DNA methylation), and activates stress signaling pathways, leading to upregulated expression of pro-inflammatory genes (such as IL-1β, TNF-α) and pro-apoptotic genes (such as Bax, caspase-3), while inhibiting the transcription of antioxidant genes (such as SOD, HO-1) and mitochondrial biogenesis genes (such as PGC-1α, TFAM) (Zhang et al., 2002; Plascencia-Villa and Perry, 2023).

In-depth analysis of reciprocal and context-dependent inter-organelle crosstalk driven by oxidative stress not only helps reveal the common pathological patterns of different neurological diseases but also provides a theoretical basis for the development of multi-target synergistic therapeutic strategies. Based on peer-reviewed literature, this review systematically elaborates the core molecular mechanisms, distinguishes well-established multi-model convergent conclusions from preliminary single-cell/single-animal speculative findings by standardized descriptive qualifiers throughout the full text, summarizes disease-specific features, and discusses potential intervention targets, aiming to provide a comprehensive reference for research in related fields.

## 2 Core mechanisms of oxidative stress-driven organelle cascade damage

**Figure 1:**
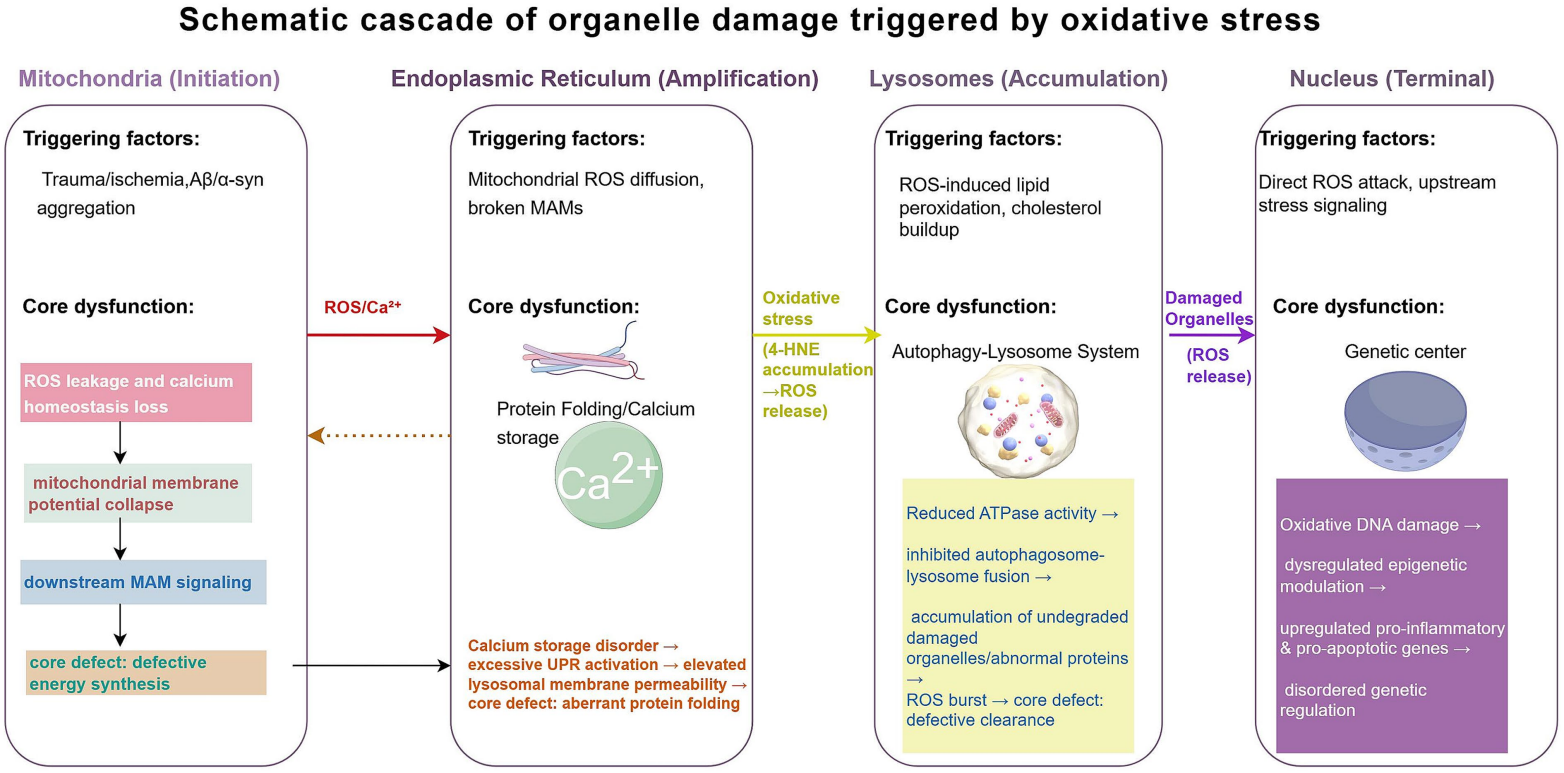
Four horizontal functional modules are sequentially arranged in the figure: mitochondria (initiation stage), endoplasmic reticulum (signal amplification stage), lysosomes (stage of impaired clearance function), and nucleus (terminal genetic damage stage). The color coding rules are as follows: solid red arrows indicate oxidative damage induced by reactive oxygen species (ROS); solid orange arrows represent dysregulated calcium signaling; solid yellow arrows refer to defective autophagic flux; solid purple arrows stand for nuclear epigenetic abnormalities and disturbed gene regulation; dashed arrows denote indirect feedback vicious cycles (By Figdraw).

It is critical to clarify before elaborating organelle injury mechanisms that oxidative stress-induced organelle damage does not follow a fixed, unidirectional mitochondria→ER → lysosome→nucleus linear sequence. The crosstalk is fully reciprocal and context-dependent. Although mitochondrial dysfunction is the most frequently observed early pathological event across most neurological disorders, primary lesions in lysosomes (e.g., LRRK2 mutation-mediated lysosomal acidification defects in PD) or MAM complexes (e.g., VAPB/SIGMAR1 mutation disrupting ER-mitochondrial tethering in ALS) can independently trigger the complete oxidative injury cascade without prior mitochondrial ROS overproduction. This bidirectional, multi-initiator property will be repeatedly reflected in each disease subsection of Section 3 (Figure 1).

### 2.1 Mitochondria: initiation and core regulation of cascade damage

MMitochondria serve as the primary targets of oxidative stress cascade damage. Their dysfunction manifests in four key aspects. These aspects include excess ROS generation, defective respiratory chain, calcium overload, and the release of pro-apoptotic factors. The manifestations vary distinctly across different pathological conditions.

Selective peroxidation of cardiolipin (CL) takes place 3 h post-TBI. CL is a unique phospholipid specific to mitochondria. Peroxidation preferentially targets CL molecules with docosahexaenoic acid (C₂₂:₆). This event emerges earlier than apoptotic markers and oxidative stress markers. Apoptotic markers include caspase-3 activation and TUNEL positivity. Oxidative stress markers include depleted glutathione and ascorbic acid. Mitochondrial electron transport chain activity drops sharply at the same time. The impaired activity refers to rotenone-sensitive NADH oxidase activity. This evidence proves mitochondrial dysfunction is an early event of TBI (Bayir et al., 2007). Meanwhile, peroxynitrite (ONOO^−^) acts as a core oxidative stress mediator. It directly injures mitochondria. The respiratory control rate (RCR) declines. State III and state V respiration are suppressed. Multiple oxidative damage markers accumulate, such as protein carbonyl (PC), 4-hydroxynonenal (4-HNE), and 3-nitrotyrosine (3-NT). Besides, broken mitochondrial membrane integrity stimulates more ROS production. A positive feedback loop is thus formed (Singh et al., 2007).

In ischemia–reperfusion injury, reverse electron transfer (RET) of mitochondrial complex I drives massive ROS release. A great quantity of succinate accumulates in brain tissue during ischemia. Reperfusion initiates succinate oxidation. This oxidation triggers RET at complex I. Bound flavin mononucleotide FMN dissociates from complex I as a result. FMN dissociation serves as the dominant ROS generator during RET and drives a biphasic shift in cellular ROS levels: transient robust ROS elevation arises from disrupted complex I electron flow upon FMN release, followed by sustained ROS reduction secondary to the loss of intact respiratory complex catalytic function (Stepanova et al., 2017). This biphasic change arises from transient FMN dissociation during complex I RET, which first triggers robust ROS generation and subsequently reduces ROS output as the complex loses structural integrity. Furthermore, the opening of mitochondrial permeability transition pore (mPTP) is a key event in ischemia–reperfusion, which can lead to loss of membrane potential, massive production of ROS, and release of cytochrome c, thus activating apoptotic pathways (Readnower et al., 2011).

In neurodegenerative diseases, mitochondrial dysfunction exhibits obvious disease specificity.

In AD, Aβ oligomers bind to mitochondrial Aβ-binding alcohol dehydrogenase (ABAD), inhibiting the activity of respiratory chain complex IV, which results in excessive ROS production and reduced ATP synthesis (supported by human post-mortem tissue and multiple transgenic AD mouse convergent evidence) (Rai et al., 2020). In PD, *α*-synuclein (*α*-syn) fibrils bind to heme in the mitochondrial respiratory chain, interfering with electron transport and leading to increased hydrogen peroxide production and loss of membrane potential (only validated in single primary dopaminergic neuron cultures and MPTP mouse models, lacking human clinical cohort verification) (Scheiblich et al., 2024). In HD, mutant huntingtin (mHTT) interacts with mitochondrial proteins (such as TIM23), disrupting protein homeostasis, reducing the activity of electron transport chain complexes II, III, and IV, and promoting mitochondrial fission by activating the GTPase activity of DRP1, thereby exacerbating ROS release (Kathiresan et al., 2025). In ALS, mutant SOD1 binds to voltage-dependent anion channel 1 (VDAC1) through its N-terminal domain, inhibiting ADP transport across the outer mitochondrial membrane, reducing energy supply, and increasing ROS levels (Argueti-Ostrovsky et al., 2024) (Figure 1).

### 2.2 Endoplasmic reticulum: calcium homeostasis imbalance and stress signal amplification

The endoplasmic reticulum participates in calcium signal regulation and protein folding through functional coupling with mitochondria. Under oxidative stress, abnormal interaction between these two organelles is a key step in the amplification of cascade damage, which is mainly characterized by disrupted calcium homeostasis and activation of the unfolded protein response (UPR).

Mitochondria-associated endoplasmic reticulum membranes (MAMs) serve as the structural basis for calcium signal transmission. In particular, the complex formed by IP3 receptor (IP3R), glucose-regulated protein 75 (GRP75), and VDAC1 mediates calcium transport from the endoplasmic reticulum to mitochondria. After TBI, mitochondrial ROS impair MAMs structure, causing dysfunction of the IP3R-VDAC1-GRP75 complex, excessive release of calcium from endoplasmic reticulum stores, and subsequent calcium overload (Thapak and Gomez-Pinilla, 2024).

In AD, Aβ oligomers exert stage-dependent bidirectional remodeling effects on MAM structure and ER-mitochondrial calcium trafficking, which explains seemingly conflicting results across published studies caused by differences in Aβ concentration, cell culture systems, and pathological staging. At the early pre-clinical compensatory pathological stage, low-dose Aβ peptide upregulates MAM assembly and elevates ER-mitochondrial contact sites as a self-protective response to stabilize calcium buffering function, as demonstrated in cellular AD models (Del Prete et al., 2017; Yu et al., 2021). After prolonged disease progression with massive accumulation of high-concentration Aβ oligomers, the IP3R–GRP75–VDAC1 tripartite complex at MAMs is disassembled, which reduces MAMs contact density and severely inhibits bidirectional ER-mitochondrial calcium shuttling (Garcia-Casas et al., 2023; García Casas et al., 2024). Meanwhile, ROS oxidatively modify IP3R and increase its opening probability, leading to abnormal calcium release from the endoplasmic reticulum (Ryan et al., 2020).

In PD, *α*-syn aggregation suppresses Mfn2 expression. It destroys MAMs integrity. Calcium transmission is impaired. Endoplasmic reticulum stress is induced accordingly (Sunanda et al., 2021).

Three core UPR pathways get activated under endoplasmic reticulum stress. They are the PERK/eIF2α, IRE1/XBP1, and ATF6 branches. PERK phosphorylates eIF2α. This suppresses overall protein synthesis. Meanwhile, it elevates pro-apoptotic proteins including ATF4 and CHOP. After TBI, oxidative stress turns on the PERK pathway. CHOP expression is upregulated. Neuronal apoptosis becomes more severe (Zhao et al., 2024). In AD, overactivated PERK/eIF2α pathway causes tau hyperphosphorylation (Uddin et al., 2020). In ALS, VAPB mutation breaks MAMs structure. It activates the IRE1-XBP1 pathway. It also boosts the secretion of pro-inflammatory factors (Chen et al., 2021).

Endoplasmic reticulum stress can worsen mitochondrial damage in return. This creates a vicious cycle. For example, ischemia–reperfusion injury causes endoplasmic reticulum calcium overload. More calcium enters mitochondria through MCU. mPTP opening is stimulated. A massive ROS release follows (Tang et al., 2025). In HD, endoplasmic reticulum stress activates IRE1. The activated IRE1 induces mitochondrial fission via the JNK pathway. Respiratory chain function is further compromised (Naia et al., 2021) (Figure 1).

### 2.3 Lysosomes: autophagic clearance defects and damage accumulation

EExcess ROS peroxidizes lysosomal membrane lipids. It inactivates acid hydrolases. It also blocks the fusion of autophagosomes and lysosomes. This process ultimately arrests autophagic degradation.

Lysosomes clear damaged organelles and abnormal proteins via the autophagy-lysosome system. Their functional integrity is critical for blocking cascade damage. Lysosomal membranes are rich in lipids and proteins. They are highly susceptible to ROS attack. Therefore, oxidative stress mainly disrupts lysosomal membrane stability and inhibits autophagic flux. This is the primary mechanism underlying oxidative stress-induced lysosomal functional defects.

Specifically after TBI, accumulated 4-HNE from lipid peroxidation disrupts lysosomal membrane permeability and triggers cytoplasmic cathepsin leakage to activate apoptosis and secondary mitochondrial injury; meanwhile, ubiquinol intervention stabilizes mitochondrial membrane potential, activates the PINK1/Parkin mitophagy cascade, improves mitochondrial morphology, lowers ROS output, and restores autophagosome-lysosome fusion to alleviate downstream apoptotic injury (Courtes et al., 2020).

In AD, cholesterol accumulation causes depletion of mitochondrial glutathione (GSH), while excessive ROS reduce lysosomal membrane potential and inhibit the fusion of autophagosomes with lysosomes (Roca-Agujetas et al., 2021). In PD, LRRK2 mutations reduce lysosomal ATPase activity, impair acidification capacity, and result in the ineffective degradation of autophagic substrates (only verified in LRRK2 G2019S transgenic mouse strains) (Sunanda et al., 2021).

Notably, obstruction of autophagic flux represents a core consequence of lysosomal functional defects, manifesting as increased autophagosome formation coupled with delayed clearance. In this context, ubiquinol pretreatment after TBI can improve mitochondrial morphology, reduce ROS production, promote the fusion of autophagosomes and lysosomes, and thereby decrease downstream apoptosis (Pierce et al., 2018).

In AD, Aβ inhibits the activity of lysosomal enzymes such as PreP, impairing the clearance of abnormal protein aggregates; simultaneously, it suppresses PINK1/Parkin-mediated mitophagy, leading to the accumulation of damaged mitochondria (Rai et al., 2020). In ALS, TBK1 mutations inhibit the phosphorylation of autophagy receptors like OPTN, hindering the ubiquitination labeling of damaged mitochondria and causing autophagic flux obstruction (Pan et al., 2023).

Furthermore, damage accumulation caused by lysosomal dysfunction further amplifies oxidative stress. For instance, in HD, mHTT binds to Beclin1 to inhibit autophagosome formation, resulting in the accumulation of damaged mitochondria and misfolded proteins that exacerbate ROS production (Severo et al., 2020). In a repeated concussion model, lysosomes fail to clear damaged mitochondria, leading to continuous ROS release and subsequent secondary oxidative damage (Severo et al., 2020) (Figure 1).

### 2.4 Nucleus: genetic material damage and abnormal gene regulation

The nucleus stores and regulates genetic information. It acts as the central target of oxidative stress cascade damage. Its dysfunction features three major manifestations. These include DNA oxidative damage, abnormal epigenetic modifications, and dysregulated expression of stress-related genes.

ROS directly attack nuclear DNA. They trigger base oxidation, such as the production of 8-hydroxydeoxyguanosine. They also induce DNA strand breaks. Within 2–72 h post-TBI, mitochondrial apoptosis-inducing factor (AIF) translocates into the nucleus. It accumulates in euchromatin regions. Massive 50 kbp DNA fragmentation occurs simultaneously (Zhang et al., 2002). In AD, ROS-triggered DNA damage activates poly ADP-ribose polymerase (PARP). PARP depletes NAD^+^. Mitochondrial function is further impaired (Zhang et al., 2017). In PD, *α*-syn aggregation generates excess ROS. These ROS oxidize nuclear DNA and activate the p53 pathway (only observed in single α-syn overexpression cell models) (Barzegar Behrooz et al., 2024).

Abnormal epigenetic modifications constitute an important mechanism by which oxidative stress impairs nuclear function, including DNA methylation, histone modification, and non-coding RNA regulation.

In AD, ROS activate DNA methyltransferase (DNMT1), leading to hypermethylation of the TIMP-2 promoter, reduced TIMP-2 expression, and aggravated extracellular matrix remodeling (Mondal et al., 2019). In HD, mHTT inhibits the transcriptional activation of PGC-1α, resulting in decreased histone acetyltransferase activity and suppressed expression of mitochondrial biogenesis genes such as TFAM (Kathiresan et al., 2025). In ALS, the G4C2 repeat transcript of C9ORF72 inhibits histone deacetylase activity, giving rise to abnormal H3K27ac modification (Rizea et al., 2024).

Oxidative stress also modulates gene expression by activating nuclear transcription factors.

Under physiological conditions, Nrf2, a key antioxidant transcription factor, binds to Keap1 in the cytoplasm. Under oxidative stress, Nrf2 dissociates and translocates into the nucleus, where it binds to antioxidant response elements (ARE) and activates the expression of HO-1, SOD, and other antioxidant genes. In TBI, tannic acid treatment reduces lipid peroxidation by activating the Nrf2/HO-1 pathway (Salman et al., 2020). In AD, Aβ inhibits Nrf2 nuclear translocation and weakens antioxidant defense (He et al., 2023).

In addition, oxidative stress activates NF-κB, a master pro-inflammatory transcription factor. It upregulates the expression of pro-inflammatory genes such as IL-1β and TNF-α, thereby exacerbating neuroinflammation (Sadhukhan et al., 2026).

In summary, oxidative stress triggers a sequential and interactive cascade injury that sequentially targets mitochondria, endoplasmic reticulum, lysosomes, and nucleus. This sequential description only reflects the most common pathological sequence in most disease models, rather than an irreversible fixed order; primary lysosomal or MAM lesions can reverse this progression. Mitochondrial dysfunction acts as the initiating event, followed by endoplasmic reticulum stress amplification, lysosomal clearance failure, and ultimately nuclear genetic and epigenetic disorders. This organelle-level cascade forms a self-reinforcing vicious cycle that promotes the progression of multiple neurological diseases (Figure 1).

## 3 Specific cascade mechanisms in different neurological diseases

### 3.1 Traumatic brain injury (TBI)

**Figure 2:**
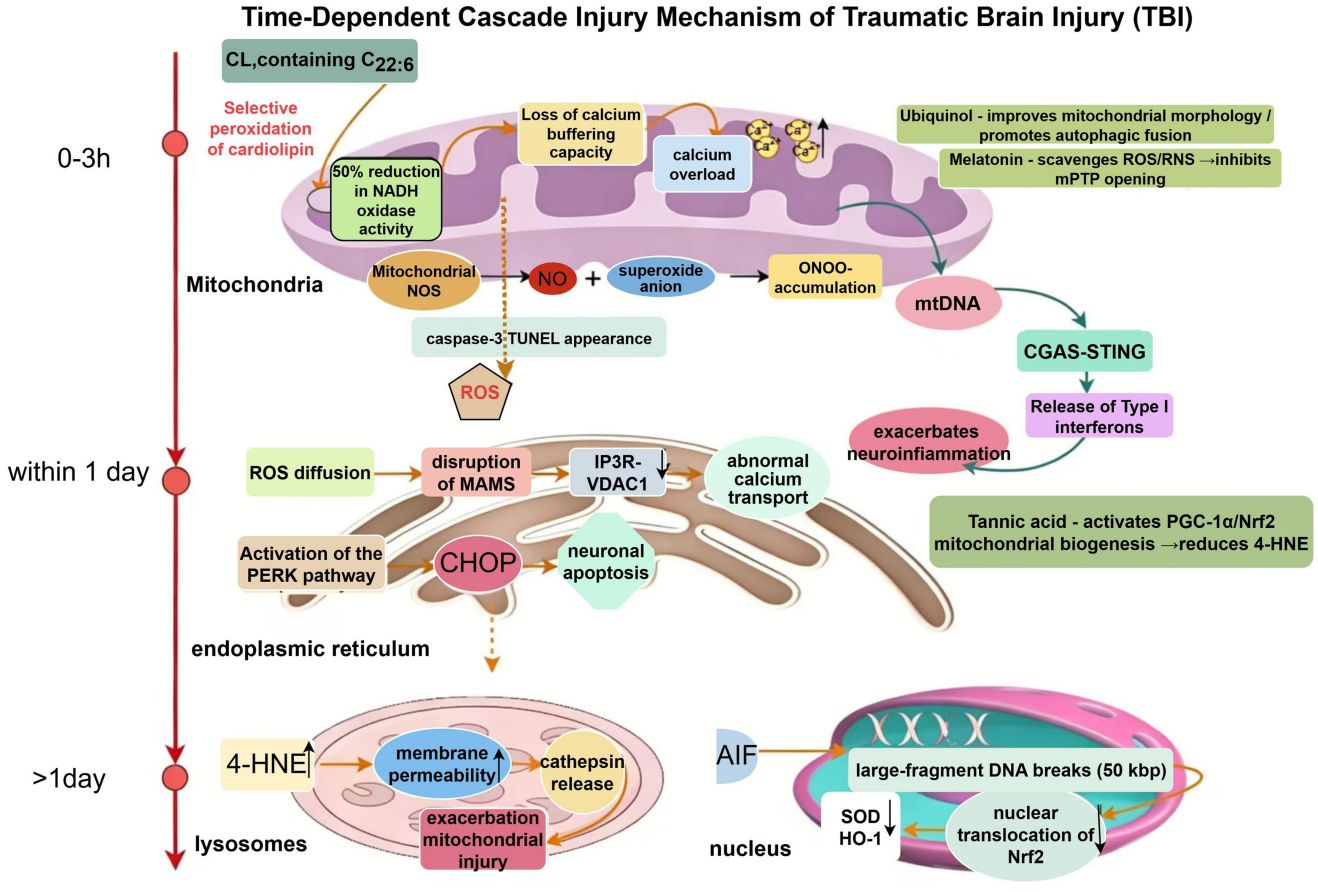
Solid red arrows illustrate a cascade of sequential reactions occurring in mitochondria, endoplasmic reticulum, lysosomes and the nucleus across three progressive stages. The meanings of various shapes and colors used in the figure are labeled within the diagram, and corresponding therapeutic interventions targeting the above damage cascades are also listed (By Figdraw).

The cascade damage of TBI has obvious time-dependent and spatial diffusion characteristics. The early stage centers on mitochondrial targeted damage. CL peroxidation reduces respiratory chain complex activity. NADH oxidase activity drops by half. Mitochondrial calcium buffering capacity weakens. Calcium overload is triggered consequently (Bayir et al., 2007; Xiong et al., 1997) (Figure 2).

One day later, oxidative stress spreads to other phospholipids, such as phosphatidylserine and phosphatidylcholine. It disturbs endoplasmic reticulum calcium homeostasis through MAMs. It activates the PERK/eIF2α pathway. It significantly upregulates CHOP expression (Zhao et al., 2024; Pandya et al., 2023) (Figure 2). Peroxynitrite (ONOO^−^) is a key mediator of oxidative stress in TBI. It forms from NO produced by mitochondrial NOS and superoxide anion. It induces protein nitration with elevated 3-NT. It also causes lipid peroxidation with accumulated 4-HNE. It directly inhibits complex I activity (Singh et al., 2007; Hill et al., 2017) (Figure 2).

In addition, mitochondria release mtDNA after TBI. The released mtDNA activates the cGAS-STING pathway. It boosts the release of type I interferons. It further exacerbates neuroinflammation (Thapak and Gomez-Pinilla, 2024) (Figure 2).

Therapeutic strategies focus on mitochondrial protection and oxidative stress inhibition. Ubiquinol pretreatment relieves mitochondrial swelling and cristae structural disorder. It also decreases serum GFAP and UCH-L1 levels (Pierce et al., 2018). Tannic acid upregulates TFAM to promote mitochondrial biogenesis. It activates the PGC-1α/Nrf2 pathway and reduces 4-HNE levels (Salman et al., 2020); melatonin directly scavenges ROS/RNS. It inhibits mPTP opening. It maintains the activity of respiratory chain complexes I and IV. It reduces cytochrome c release (Pandi-Perumal et al., 2013) (Figure 2).

### 3.2 Alzheimer’s disease (AD)

**Figure 3:**
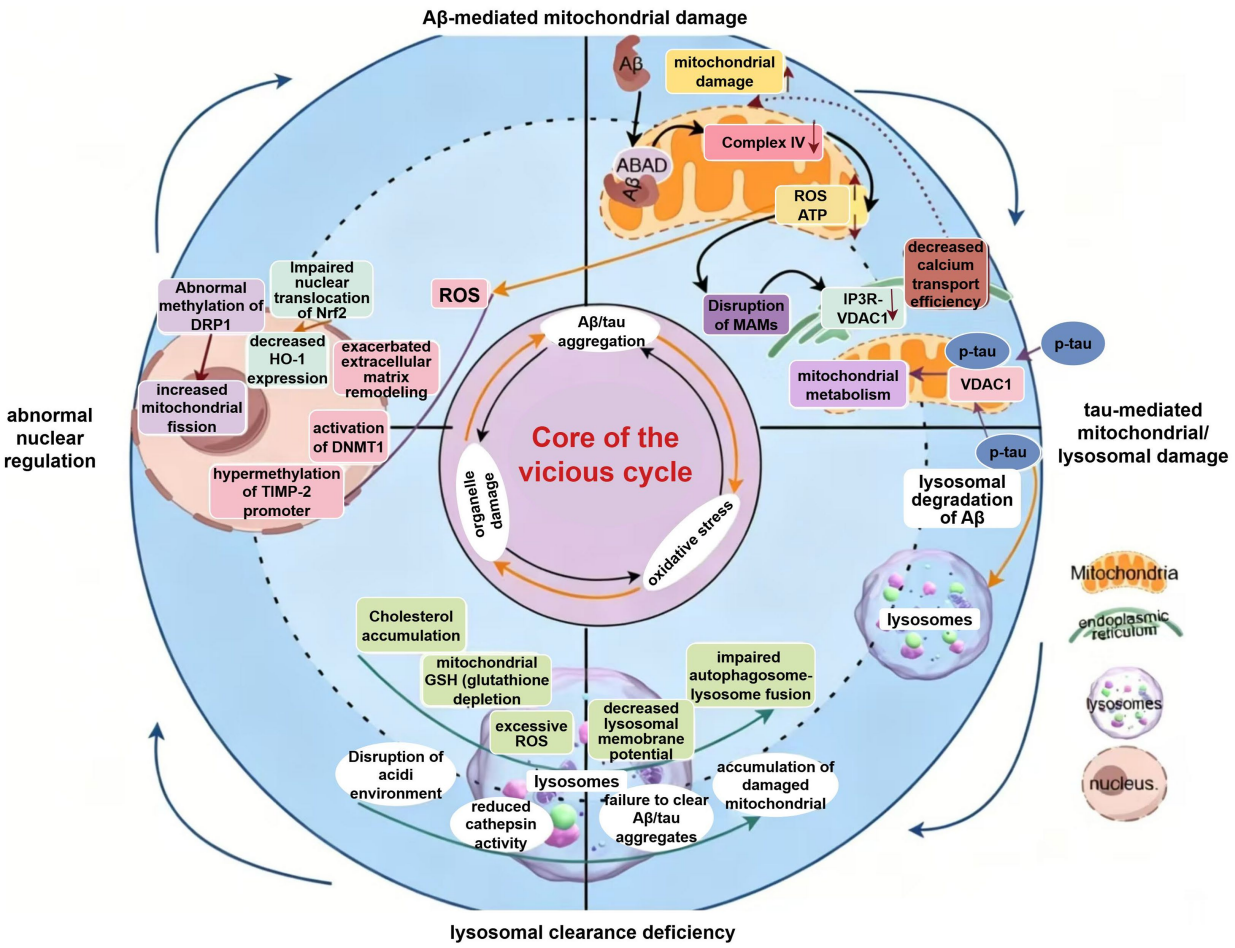
This schematic diagram uses fan-shaped graphics to illustrate the core vicious cycle formed by *β*-amyloid/tau protein aggregation, oxidative stress and organelle damage in Alzheimer’s disease, which is indicated by the outer solid arrows. Distinct mechanistic pathways are distinguished by different colors, and the name and implication of each element are labeled directly on the corresponding element (By Figdraw).

TThe cascade damage of AD is driven by Aβ and tau pathology. It forms a vicious cycle of “protein aggregation-oxidative stress-organelle damage.” Aβ oligomers bind to mitochondria via ABAD. They suppress complex IV activity. ROS production rises sharply. They also disrupt MAMs structure. IP3R-VDAC1 co-localization declines. Calcium transport efficiency drops drastically (Rai et al., 2020; Ryan et al., 2020). Hyperphosphorylated tau interacts with VDAC1. It hinders mitochondrial metabolism. It also blocks lysosomal clearance of Aβ (Quntanilla and Tapia-Monsalves, 2020) (Figure 3).

Lysosomal functional defects exert dual effects in AD. On one hand, impaired acidic microenvironment lowers cathepsin activity. Cells cannot eliminate Aβ and tau aggregates. On the other hand, blocked autophagic flux accumulates damaged mitochondria. These mitochondria continuously release ROS (Roca-Agujetas et al., 2021; Jurcau, 2021). At the nuclear level, ROS trigger abnormal methylation of the DRP1 promoter. This event accelerates mitochondrial fission. Meanwhile, Nrf2 nuclear translocation is suppressed. The antioxidant gene HO-1 is downregulated (He et al., 2023) (Figure 3).

Intervention strategies include: 40 Hz light therapy restores the activity of mitochondrial complexes I/IV, upregulates mitoBKCa channel function, and reduces nuclear DNA damage (only validated in 5XFAD single transgenic mouse model) (Barzegar Behrooz et al., 2024); luteolin activates PPARγ, promotes PGC-1*α*-mediated mitochondrial biogenesis, and increases IDE expression to clear Aβ (He et al., 2023); *Ginkgo biloba* extract reduces MAO activity, increases SOD and GSH-Px levels, and decreases 8-OHdG accumulation (Xia et al., 2024) (Figure 3).

### 3.3 Parkinson’s disease (PD)

**Figure 4:**
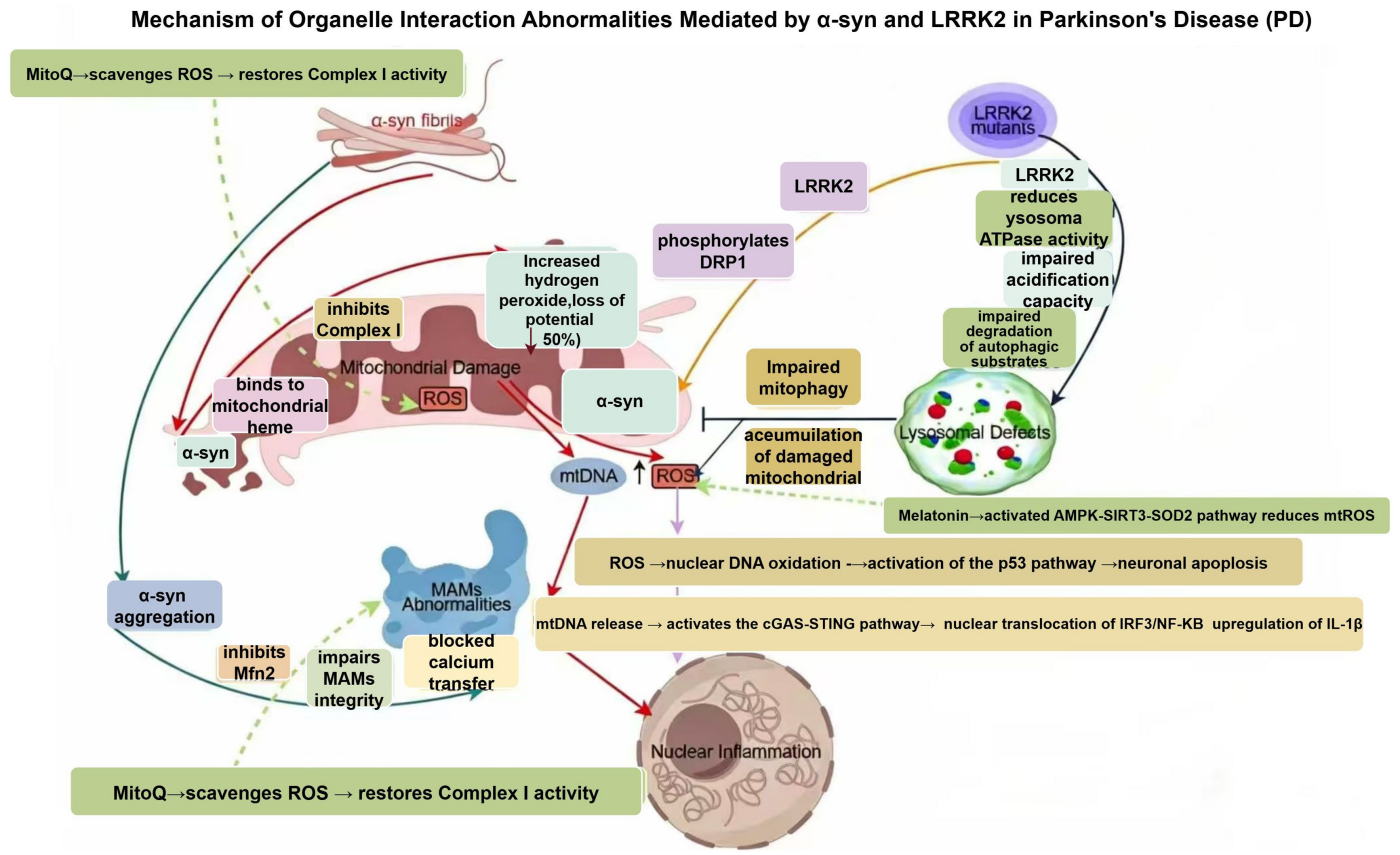
In this schematic diagram, solid lines denote the transmission of mechanistic pathways, with the name and implication of each element labeled directly on the element itself; dashed lines stand for target interventions, and lines of different colors correspond to distinct pathways (By Figdraw).

The cascade damage of PD is centered on *α*-syn aggregation and mitochondrial dysfunction. α-syn fibrils bind to mitochondrial heme, inhibiting the activity of complex I, leading to a significant increase in hydrogen peroxide production and a halved membrane potential (Scheiblich et al., 2024). LRRK2 mutation promotes mitochondrial fission by phosphorylating DRP1, and simultaneously reduces lysosomal ATPase activity, sharply impairing acidification capacity, this lysosomal primary defect precedes overt mitochondrial injury, matching the bidirectional cascade caveat proposed in Section 2 opening and inhibiting mitophagy (Sunanda et al., 2021; Sadhukhan et al., 2026) (Figure 4).

Abnormal MAMs structure is a key feature of PD: α-syn aggregation reduces the number of MAMs, decreases the co-localization of IP3R and mitochondria, significantly downregulates calcium transport, and triggers endoplasmic reticulum stress; DJ-1 deficiency affects the IP3R3-GRP75-VDAC1 complex, exacerbating calcium imbalance (Sunanda et al., 2021). In the nucleus, mtDNA release activates the cGAS-STING pathway, increases IRF3 and NF-κB nuclear translocation, and upregulates the expression of the pro-inflammatory factor IL-1β (Sadhukhan et al., 2026) (Figure 4).

Targeted therapies include: Mitochondria-targeted antioxidant MitoQ scavenges ROS and restores complex I activity (Silachev et al., 2015); Pridopidine activates the Sigma-1 receptor, restores MAMs structure, and increases mitochondrial elongation (Naia et al., 2021); melatonin reduces mtROS through the Akt-SIRT3-SOD2 pathway and inhibits apoptosis (Long et al., 2020) (Figure 4).

### 3.4 Huntington’s disease (HD)

The cascade damage of HD is triggered by mHTT aggregation. It mainly targets striatal neurons. mHTT binds to TIM23. This interaction disrupts mitochondrial protein import. Complex II and III activity decline markedly. DRP1 GTPase activity is activated. Mitochondrial fission increases dramatically (Kathiresan et al., 2025; Ambekar et al., 2021). Abnormal MAMs structure reduces calcium transport. It activates endoplasmic reticulum stress. PERK phosphorylation levels rise significantly (Naia et al., 2021).

Lysosomal autophagy defects aggravate HD pathology. mHTT binds Beclin1 and blocks autophagosome formation. p62 accumulates in large quantities. HDAC6 inhibition blocks autophagic flux. Clearance of damaged mitochondria is delayed (Pan et al., 2023; Pantiya et al., 2020). In the nucleus, mHTT suppresses PGC-1α. It lowers expression of mitochondrial biogenesis genes NRF1/2. It also induces abnormal histone H3K27ac modification. Antioxidant gene expression is inhibited accordingly (Kathiresan et al., 2025).

Therapeutic strategies are listed as follows. Sigma-1 receptor agonists restore calcium transport in MAMs and improve mitochondrial respiratory function (Naia et al., 2021). C60 fullerene facilitates Nrf2 nuclear accumulation. It upregulates GCLC and GSTP, and recovers the balance of GSH/GSSG. The 3-nitropropionic acid (3-NPA) model is a classic striatal injury model induced by mitochondrial complex II inhibition. It recapitulates HD-like pathological phenotypes. Regulating iron metabolism relieves lipid peroxidation (Gonchar et al., 2021).

### 3.5 Amyotrophic lateral sclerosis (ALS)

TThe cascade damage of ALS involves multiple gene mutations. It presents genetic heterogeneity. Mutant SOD1 binds to VDAC1. This binding suppresses ADP transport. ATP production drops by one third. ROS generation is elevated (Argueti-Ostrovsky et al., 2024). TDP-43 aggregation disturbs mitochondrial dynamics. It blocks axonal transport. It also hinders the recruitment of autophagy receptor OPTN (Zuo et al., 2021). The poly-GR protein from C9ORF72 binds to mitochondrial ribosomes. It suppresses oxidative phosphorylation (Rizea et al., 2024).

VAPB and SIGMAR gene mutation-induced MAM structural breakdown acts as an initial pathological trigger before obvious mitochondrial functional impairment, consistent with the non-linear organelle crosstalk framework.

Abnormal MAMs function is a shared hallmark of ALS. VAPB mutation breaks ER-mitochondrial connections. Calcium transport capacity is cut in half (Chen et al., 2021). SIGMAR1 mutation causes IP3R degradation. It further activates endoplasmic reticulum stress (Chen et al., 2021). TBK1 and OPTN mutations block lysosomal autophagic flux. Damaged mitochondria and protein aggregates build up (Pan et al., 2023). Inside the nucleus, NF-κB activation boosts pro-inflammatory factor expression. Less Nrf2 translocates into the nucleus. Antioxidant defense capacity is weakened (Russo et al., 2025).

Interventions are listed as follows. Polyphenols target ferroptosis. They suppress lipid peroxidation (Russo et al., 2025). NAD^+^ precursor NR activates the SIRT1/PGC-1α pathway. It improves mitochondrial performance (Obrador et al., 2021). Melatonin regulates autophagy. It alleviates TDP-43 aggregation (Luo et al., 2020).

Collectively, oxidative stress-driven organelle cascade damage has unique disease-specific traits in TBI, AD, PD, HD, and ALS. These diseases have distinct pathogenic initiators. Yet they share core pathological manifestations. The shared features include mitochondrial dysfunction, disrupted inter-organelle crosstalk, impaired lysosomal autophagy, and abnormal nuclear regulation. The combined commonalities and specificities lay a solid theoretical foundation for precise targeted therapy.

## 4 Therapeutic strategies and translational challenges

Current therapeutic strategies targeting oxidative stress-mediated inter-organelle crosstalk show favorable neuroprotective effects in preclinical models (*in vitro* and animal studies). However, clinical translation faces substantial challenges, including poor organelle-targeting specificity, insufficient blood–brain barrier penetration, short half-life, and failure to disrupt established reciprocal injury cycles. Conventional antioxidant therapies have shown limited clinical efficacy due to their non-selective nature and inability to reverse bidirectional organelle damage (Tables 1, 2).

**Table 1:** Summary of therapeutic strategies targeting oxidative stress-mediated inter-organelle crosstalk.

Compound	Target organelle	Mechanism of action	Evidence level	Limitations	Clinical trial phase
Ubiquinol	Mitochondria	Improves mitochondrial morphology and promotes autophagy	Preclinical (animal)	Poor blood–brain barrier penetration, short circulating half-life	No human clinical trials initiated
Tannic acid	Mitochondria/Nucleus	Activates PGC-1α/Nrf2/HO-1 signaling pathway	Preclinical (animal)	Low oral bioavailability	Preclinical research only
Melatonin	Mitochondria/Endoplasmic reticulum/Lysosomes	Scavenges ROS, inhibits mPTP opening, regulates autophagy	Preclinical	No specific mitochondrial enrichment capacity	Phase II completed for sleep disorders; no dedicated trials for neurological diseases
MitoQ	Mitochondria	Scavenges mitochondrial ROS, restores complex I function	Preclinical	Systemic side effects at high doses	Phase I safety trial for nervous system completed
Pridopidine	MAMs	Restores endoplasmic reticulum-mitochondria contact sites	Preclinical	Slow distribution in central nervous tissues	Phase III ongoing for Huntington’s disease
Mitochondria-targeted resveratrol	Mitochondria	Activates SIRT3/PGC-1α pathway	Preclinical	Rapid hepatic metabolism	Preclinical
Spermidine	Lysosomes/Mitochondria	Induces PINK1/Parkin-mediated mitophagy	Preclinical	Low intestinal absorption rate	Phase II recruiting for cognitive decline

**Table 2:** Disease-specific triggers, affected organelles, biomarkers, therapeutic targets and evidence strength.

Disease	Primary triggers	Main organelles	Key biomarkers	Therapeutic targets	Evidence strength
TBI	Mechanical trauma	Mitochondria > ER > Lysosomes	4-HNE, 3-NT, GFAP	Mitochondrial protection, ROS scavenging	Strong (animal)
AD	Aβ/tau aggregation	MAMs, Lysosomes, Nucleus	8-OHdG, p-tau	Nrf2, MAMs, Mitophagy	Moderate
PD	α-syn, LRRK2	Mitochondria, Lysosomes, MAMs	α-syn, mtROS	Complex I, LRRK2, MAMs	Moderate
HD	mHTT aggregation	Mitochondria, ER	mHTT, p62	MAMs, Autophagy	Moderate
ALS	SOD1/TDP43/C9ORF72	MAMs, Mitochondria, Lysosomes	TDP-43, SOD1	MAMs, Autophagy	Moderate

## 5 Conclusion and outlook

Oxidative stress drives interconnected, bidirectional, and disease-specific inter-organelle crosstalk rather than a rigid unidirectional mitochondria→ER → lysosome→nucleus cascade. Mitochondria frequently act as an early functional hub, but the pathological cascade is reciprocal, cell-type-specific, disease-stage-dependent, and can be initiated by multiple primary triggers beyond mitochondrial ROS, including lysosomal dysfunction, MAM disruption, ER stress, or nuclear/RNA pathology (Ghosh et al., 2022, 2023; Ghosh and Kumar, 2024). In TBI, mitochondrial cardiolipin peroxidation represents a consistent early event; in PD, LRRK2-mediated lysosomal defects or *α*-synuclein aggregation can precede overt mitochondrial injury; in ALS, VAPB/SIGMAR1-related MAM abnormalities or C9ORF72 nuclear repeat-driven RNA toxicity can serve as primary initiators; in HD, mHTT-induced MAM and mitochondrial impairment are interdependent.

Current evidence supports the existence of interconnected organelle dysfunction across neurological diseases but lacks direct, *in vivo*, time-resolved experimental data from a single unified model system that definitively establishes a universal linear sequence of organelle damage. Most existing findings derive from distinct disease models, different time points, and varied experimental systems, limiting the ability to confirm a strict temporal order. While mitochondrial dysfunction is often observed early in many contexts, it does not represent an absolute or universal starting point across all disease mechanisms.

Future research directions should focus on several key priorities: (1) Applying advanced super-resolution imaging and in vivo time-lapse techniques to capture dynamic organelle interactions and define spatiotemporal crosstalk in living neurons; (2) Identifying disease-specific early biomarkers derived from organelle damage (e.g., mtDNA fragments, oxidized lipids, lysosomal hydrolases) to enable early diagnosis and intervention; (3) Developing highly specific organelle-targeted delivery systems to improve the efficacy and safety of antioxidant and neuroprotective agents; (4) Designing combinatorial therapeutic strategies that simultaneously target multiple interconnected nodes to disrupt reciprocal injury loops; (5) Constructing clinically relevant disease models (e.g., patient-derived organoids) to accelerate translational research and bridge preclinical findings to clinical applications.

A comprehensive understanding of oxidative stress-mediated inter-organelle crosstalk will advance our mechanistic insights into neurological diseases and facilitate the development of precise, multi-target therapeutic interventions, ultimately improving patient prognosis.

## Glossary

TBI: Traumatic Brain Injury
PD: Parkinson’s Disease
ALS: Amyotrophic Lateral Sclerosis
OXPHOS: Oxidative Phosphorylation
CL: Cardiolipin
ONOO^−^: Peroxynitrite
PC: Protein Carbonyl
3-NT: 3-Nitrotyrosine
FMN: Flavin Mononucleotide
Aβ: Amyloid β
α-syn: α-Synuclein
TIM23: Translocase of Inner Mitochondrial Membrane 23
SOD1: Superoxide Dismutase 1
MAMs: Mitochondria-Associated ER Membranes
GRP75: Glucose-Regulated Protein 75
PERK: Protein Kinase R-Like Endoplasmic Reticulum Kinase
IRE1: Inositol-Requiring Enzyme 1
ATF6: Activating Transcription Factor 6
CHOP: C/EBP Homologous Protein
LRRK2: Leucine-Rich Repeat Kinase 2
DJ-1: Protein DJ-1
PINK1: PTEN-Induced Kinase 1
p62: Sequestosome 1
TBK1: TANK-Binding Kinase 1
8-OHdG: 8-Hydroxy-2’-Deoxyguanosine
PARP: Poly ADP-Ribose Polymerase
DNMT1: DNA Methyltransferase 1
PGC-1α: Peroxisome Proliferator-Activated Receptor Gamma Coactivator 1*α*
NRF1/2: Nuclear Respiratory Factor 1/2
Nrf2: Nuclear Factor Erythroid 2-Related Factor 2
ARE: Antioxidant Response Element
NF-κB: Nuclear Factor Kappa-Light-Chain-Enhancer of Activated B Cells
TNF-α: Tumor Necrosis Factor-α
caspase-3: Cysteinyl Aspartate-Specific Protease 3
IRF3: Interferon Regulatory Factor 3
UCH-L1: Ubiquitin C-Terminal Hydrolase L1
Pridopidine: Pridopidine
VAPB: Vesicle-Associated Membrane Protein-Associated Protein B
C9ORF72: Chromosome 9 Open Reading Frame 72
IDE: Insulin-Degrading Enzyme
GSH: Glutathione
MAO: Monoamine Oxidase
GSTP: Glutathione S-Transferase Pi
3-NPA: 3-Nitropropionic Acid
SIRT3: Sirtuin 3
AD: Alzheimer’s Disease
HD: Huntington’s Disease
ROS: Reactive Oxygen Species
ATP: Adenosine Triphosphate
C₂₂:₆: Docosahexaenoic Acid
RCR: Respiratory Control Rate
4-HNE: 4-Hydroxynonenal
RET: Reverse Electron Transport
mPTP: Mitochondrial Permeability Transition Pore
ABAD: Aβ-Binding Alcohol Dehydrogenase
mHTT: Mutant Huntingtin
DRP1: Dynamin-Related Protein 1
VDAC1: Voltage-Dependent Anion Channel 1
IP3R: Inositol 1,4,5-Trisphosphate Receptor
UPR: Unfolded Protein Response
eIF2α: Eukaryotic Initiation Factor 2*α*
XBP1: X-Box Binding Protein 1
ATF4: Activating Transcription Factor 4
MCU: Mitochondrial Calcium Uniporter
Mfn2: Mitofusin 2
PreP: Presequence Protease
Beclin1: Beclin 1
HDAC6: Histone Deacetylase 6
OPTN: Optineurin
AIF: Apoptosis-Inducing Factor
NAD^+^: Nicotinamide Adenine Dinucleotide
TIMP-2: Tissue Inhibitor of Metalloproteinases 2
TFAM: Mitochondrial Transcription Factor A
H3K27ac: Histone H3 Lysine 27 Acetylation
Keap1: Kelch-Like ECH-Associated Protein 1
HO-1: Heme Oxygenase-1
IL-1β: Interleukin-1β
Bax: Bcl-2-Associated X Protein
cGAS-STING: Cyclic GMP-AMP Synthase-Stimulator of Interferon Genes
GFAP: Glial Fibrillary Acidic Protein
MitoQ: Mitochondria-Targeted Antioxidant Mitoquinone
SIGMAR1: Sigma-1 Receptor
TDP-43: TAR DNA-Binding Protein 43
mitoBKCa: Mitochondrial Large-Conductance Calcium-Activated Potassium Channel
PPARγ: Peroxisome Proliferator-Activated Receptor Gamma
GSH-Px: Glutathione Peroxidase
GCLC: Glutamate-Cysteine Ligase Catalytic Subunit
GSSG: Glutathione Disulfide
NR: Nicotinamide Riboside
